# Oximetry and neonatal examination for the detection of critical congenital heart disease: a systematic review and meta-analysis

**DOI:** 10.12688/f1000research.17989.1

**Published:** 2019-03-01

**Authors:** Hernán Camilo Aranguren Bello, Dario Londoño Trujillo, Gloria Amparo Troncoso Moreno, Maria Teresa Dominguez Torres, Alejandra Taborda Restrepo, Alejandra Fonseca, Nestor Sandoval Reyes, Cindy Lorena Chamorro, Rodolfo José Dennis Verano

**Affiliations:** 1 Research Department, Fundación Cardioinfantil – Instituto de Cardiología, Bogotá, Colombia; 2 Eje de Salud Pública, Fundación Santa Fe de Bogotá, Bogotá, Colombia; 3 Neonatal Intensive Care Unit, Fundación Cardioinfantil - Instituto de Cardiología, Bogotá, Colombia; 4 Institute of Congenital Heart Disease, Fundación Cardioinfantil – Instituto de Cardiología., Bogotá, Colombia; 5 Escuela de Medicina y Ciencias de la Salud, Universidad del Rosario, Bogotá, Colombia

**Keywords:** Oximetry, screening, critical congenital heart disease, specificity, sensitivity, physical examination, newborn

## Abstract

**Background: **Undiagnosed congenital heart disease in the prenatal stage can occur in approximately 5 to 15 out of 1000 live births; more than a quarter of these will have critical congenital heart disease (CCHD). Late postnatal diagnosis is associated with a worse prognosis during childhood, and there is evidence that a standardized measurement of oxygen saturation in the newborn by cutaneous oximetry is an optimal method for the detection of CCHD. We conducted a systematic review of the literature and meta-analysis comparing the operational characteristics of oximetry and physical examination for the detection of CCHD.

**Methods: **A systematic review of the literature was conducted on the following databases including published studies between 2002 and 2017, with no language restrictions: Pubmed, Science Direct, Ovid, Scopus and EBSCO, with the following keywords: oximetry screening, critical congenital heart disease, newborn OR oximetry screening heart defects, congenital, specificity, sensitivity, physical examination.

**Results: **A total of 419 articles were found, from which 69 were selected based on their titles and abstracts. After quality assessment, five articles were chosen for extraction of data according to inclusion criteria; data were analyzed on a sample of 404,735 newborns in the five included studies. The following values were found, corresponding to the operational characteristics of oximetry in combination with the physical examination: sensitivity: 0.92 (CI 95%, 0.87-0.95), specificity: 0.98 (CI 95%, 0.89-1.00), for physical examination alone sensitivity: 0.53 (CI 95%, 0.28-0.78) and specificity: 0.99 (CI 95%, 0.97-1.00).

**Conclusions: **Evidence found in different articles suggests that pulse oximetry in addition to neonatal physical examination presents optimal operative characteristics that make it an adequate screening test for detection of CCHD in newborns, above all this is essential in low and middle-income settings where technology medical support is not entirely available.

## Introduction

Congenital Heart Disease (CHD) in the prenatal stage can affect approximately 5 to 15 out of 1,000 live births ^1^. A significant proportion of these cases will suffer from critical congenital heart disease (CCHD) ^2^ requiring surgical treatment of intervention before the first year of life, as a late postnatal diagnosis is associated with a worse prognosis, a higher number of hospital admissions, long stays in hospital, and consequently, increased costs during childhood ^3^.

The accuracy of cutaneous oximetry as a noninvasive measure to detect arterial oxygen saturation (SaO _2_) has been studied for more than two decades in neonatology, and its importance in the care of patients with respiratory and cardiovascular compromise has been recognized ^4^. Evidence shows that the standardized, systematic measurement of SaO _2_ in newborns (NB) through transcutaneous oximetry may be a safe method with satisfactory operating characteristics in the detection of CCHD ^5– 10^. A systematic literature review (SLR) assessing the use of oximetry in the screening of CHD in NB showed that oximetry is a highly specific technology, with a very low level of false positive results, in the detection of congenital heart defects in NB ^5^. Likewise, a meta-analysis showed similar findings ^6^, highlighting the low rate of false-positive results in oximetry, primarily when the screening was conducted after the 24 hours following birth. It is worth highlighting that there are no recent studies of this type which include the evidence available on oximetry screening in the newborn for the detection of CCHDs.

Together with the use of oximetry, the role of the neonatal physical examination in the detection of CCHD has been studied ^7, 8^, revealing that NBs with CCHDs have been discharged without a timely diagnosis. This fact has been reported in the literature as relevant, suggesting that the neonatal physical examination fails to detect almost half of the cases of NB with CCHDs ^8^. The above added to the low sensitivity and a high rate of false positives in the neonatal physical examination, has aroused more interest in including oximetry in CCHD screening, and it has been found that CCHD screening has a higher sensitivity when combining the neonatal physical examination and oximetry as compared with the individual use of any of these two methods ^9^.

Evidence has shown that CCHD screening through oximetry may have optimal operating characteristics ^10– 12^ that may allow for the identification of cases that would otherwise be impossible to detect; the above may allow as well for the implementation of national screening programs for early detection of CCHD, a step that would have an impact in the reduction of neonatal mortality and the costs associated with the assistance to complications deriving from late diagnosis ^3^.

The purpose of this review was to define the operating characteristics of oximetry combined with physical examination in the detection of CCHD in NB younger than 37 weeks without suspicion or prior diagnosis of CCHDs.

## Methods

This review followed the criteria for reporting systematic literature reviews and meta-analysis as defined by the PRISMA strategy ^13^. A completed PRISMA checklist is provided on OSF ^14^.

### Population type

Newborns born after 37 weeks who underwent screening with oximetry, and who were analyzed for operative characteristics (sensitivity and specificity) were included. Studies on NB requiring neonatal intensive care or enduring infectious processes at birth were excluded.

### Interventions

The studies selected compared cutaneous oximetry screening and physical examination with physical examination alone.

### Outcomes

Newborns with CCHDs (undiagnosed in the prenatal stage), who are diagnosed early (at birth in the hospital) and not late (after hospital discharge at birth). CCHDs that can be diagnosed by screening include the following 12 CCHDs: interrupted aortic arch, coarctation of the aorta, dextro-transposition of the great arteries, double outlet right ventricle, Ebstein’s anomaly, hypoplastic left heart syndrome, pulmonary atresia, single ventricle, tetralogy of Fallot, total anomalous venous return, tricuspid atresia and truncus arteriosus.

### Literature search

**Figure 1.**
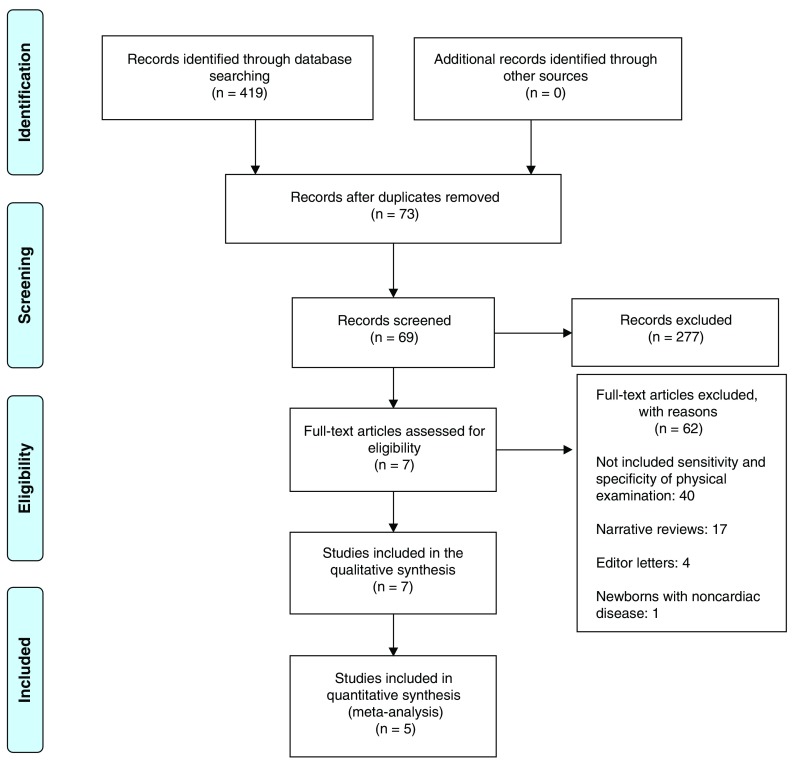
PRISMA flow diagram ^13^.

An SLR was conducted on the following databases: Pubmed, Science Direct, Ovid, Scopus, and EBSCO, with the following keywords: oximetry screening, critical congenital heart disease, newborn OR oximetry screening, heart defects, congenital, specificity, sensitivity, physical examination. Cohort and case and control observational studies were included, as well as cross-sectional studies and prospective multicenter studies published between January 2002 and December 2017, with no language restrictions ( Figure 1).

### Selection of studies and quality assessment

The selection and extraction of information, as well as the quality assessment of the articles, was performed independently by a revisor, considering the criteria of eligibility, and evaluating the bias risk, as well as the quality criteria adjusted to every type of study. Studies complying with more than 60% of the quality criteria were selected; the selection was made utilizing the STARD 2015 checklists ^15^ for diagnostic test studies, and STROBE ^16^ for observational studies. The methodological quality was also assessed employing the criteria included in the QUADAS-2 instrument ^17^.

### Data extraction

The following data related with the characteristics of the study and the outcomes of interest were selected from the studies selected: author, year, type of study, sample size, screening age, cut-off point, false positives, false negatives, positive predicting value, negative predicting value, sensitivity, and specificity.

To assess the bias risk, the following data were extracted: design of the study, blinding (in case it applies to the study of interest), losses to follow-up, outcome reports, contamination risk, and any other aspect affecting validity according to the type of the study.

The extraction was conducted for each one of the studies selected for meta-analysis, both for the physical examination and oximetry: sensitivity, specificity, predictive values, false positives, false negatives, true positives, and true negatives. In the cases where some of these data were not specified in the studies, an approximate calculation of the missing data was performed using RevMan 5.3 (Review Manager RevMan program calculator [Computer program] Version 5.3. Copenhagen: The Nordic Cochrane Centre, The Cochrane Collaboration, 2014) and a meta-analysis of diagnostic tests was performed using the STATA software version 12 (StataCorp, College Station, Texas). Confidence intervals were calculated utilizing the Midas ^18^ and metandi ^19^ programs using STATA; likelihood ratios and heterogeneity values utilizing I ^2^ statistic calculation ^20^, and publication bias probability assessment through Deeks’ asymmetry test ^21^, the most highly recommended current strategy to assess publication bias ^22^

A statistical heterogeneity assessment was conducted using the I ^2^ statistic calculation, which describes the total variation percentage among the studies, which may be attributed to heterogeneity and not chance ^20^. I ^2^ may take values between 0% and 100%, where 0% is the absence of heterogeneity observed; values above 50% suggest substantial heterogeneity. The advantage of I ^2^ calculation lies in the fact that it does not depend on the number of studies in the meta-analysis ^20^.

## Results

### Article selection and quality assessment

A total of 419 papers were identified as follows ( Figure 1): 111 in Pubmed, 76 in EBSCO, 104 in Ovid, 118 in Science Direct and 10 in Scopus; 73 were duplicates, and 69 papers were selected for full-text revision by their title and abstract review. Out of these, 40 papers were excluded, as they did not include the sensitivity or specificity of the physical examination; 17 papers were excluded because they were narrative literature reviews; four papers were letters to the editor, and one paper included newborns with non-cardiac pathologies. After verifying compliance with the inclusion criteria, seven articles were included in the final analysis for quality assessment ( Table 1 and Table 2).

**Table 1.** Characteristics of the studies included.

Author	Year	Type of study	Sample size	Screening age	Cut-off point	False positives physical examination + oximetry	False negatives physical examination + oximetry	Positive predictive value physical examination + oximetry	Negative predictive value physical examination + oximetry
**Zhao** ^24^	2014	Observational prospective multicenter study	122.378	6–72 hours	Oxygen saturation between 90–95 %	2–9 %	10	3.8 %	99 %
**De Wahl-Granelli A** ^9^	2009	Cohort study	39.821	16 hours before discharge	Oxygen saturation < 95 %	2.09 %	5	2.92 %	99.97 %
**Hu Xiao** ^28^	2017	Observational prospective multicenter study	167.190	6–72 hours	Oxygen saturation between 90–95 %	1.2 %	2	2.1 %	100 %
**Meberg** ^29^	2008	Observational prospective multicenter study	50.008	24 hours	Oxygen saturation < 95 %	0.6 %	Not reported	9.6 %	99.9 %
**Saxena** ^23^	2015	Cross- sectional study	19009	48 hours	Oxygen saturation < 95 %	6361	4	0.3 %	99.9 %
**Oakley** ^30^	2014	Observational prospective study	6329	24–36 hours	Oxygen saturation < 95 %	Not reported	Not reported	50 %	99 .9 %

**Table 2.** Characteristics of the studies included.

Author	Year	Sensitivity of oximetry	Specificity of oximetry	Sensitivity of physical examination	Specificity of physical examination	Sensitivity oximetry + physical examination	Specificity oximetry + physical examination
**Zhao** ^24^	2014	83.6 %	99.7 %	77.4 %	97.3%	93.2 %	97.1 %
**De Wahl-Granelli A** ^9^	2009	62.07 %	99.82 %	62.05 %	98.07 %	82.76 %	97.88 %
**Hu Xiao** ^28^	2017	77.3 %	99.8 %	75 %	99 %	95.5 %	98.8 %
**Meberg** ^29^	2008	77.1 %	99.4 %	Not reported	Not reported	88.6 %	99.4 %
**Saxena** ^23^	2015	84.6 %	68.3 %	11.5 %	97.2 %	84.6 %	66.5 %
**Oakley** ^30^	2014	87.5 %	Not reported	37.5 %	Not reported	87.5 %	99.8 %

**Figure 2.**
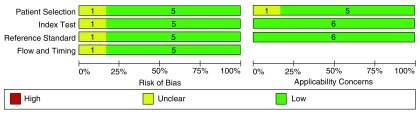
Quality assessment and bias risk according to the QUADAS-2 tool criteria for diagnostic test studies.

**Figure 3.**
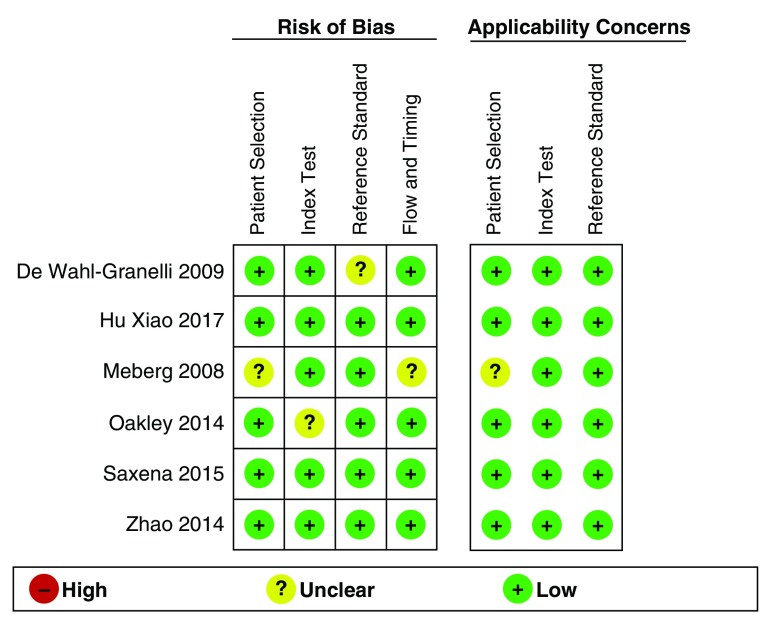
Bias risk and aspects associated with the applicability of every study included.

For every article selected, the 12 CCHDs previously mentioned were considered as outcomes of interest. Following the quality assessment, a low risk of bias was observed in individual studies due to compliance with the four criteria described in five of the articles ( Figure 2). The echocardiogram was applied as a pattern of reference in all reviews, and its results were assessed independently from the result of the oximetry screening in two of the papers ^23, 24^. These were considered, as the remaining reviews included echocardiogram only for newborns who showed alterations in the oximetry screening. It is important to mention that in most reviews no aspects were found that might hinder the applicability of the screening, both when selecting the population participating in the study and when performing the screening of the reference pattern. ( Figure 3)

Once the application of the quality criteria was performed, seven articles were selected for data extraction; one article was excluded after conducting the quality assessment; one of the articles complying with quality criteria was excluded, as it did not include comparative data between the physical examination and oximetry. Finally, five articles were selected for the meta-analysis.

### Data extracted

**Figure 4.**
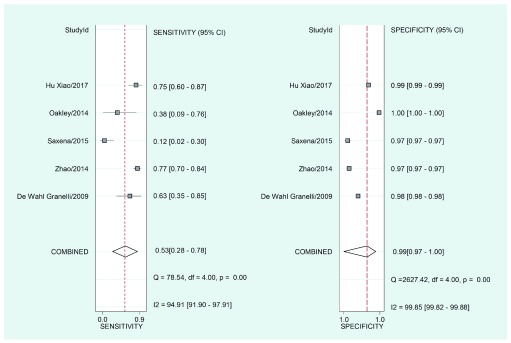
Joint sensitivity and specificity "forest plot" for the physical examination as a screening test to detect critical congenital heart disease in asymptomatic newborns.

**Figure 5.**
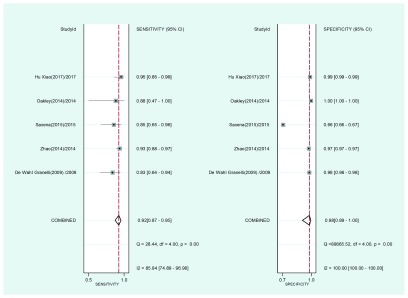
Joint “Forest plot” for sensitivity and specificity for the physical examination with oximetry as a screening test to detect critical congenital heart disease in asymptomatic newborns.

Figure 4 shows the grouped values corresponding to the operative characteristics of the physical examination: sensitivity: 0.53 (95% confidence interval (95%CI), 0.28-0.78), specificity: 0.99 (95%CI, 0.97-1.00). Upon adding the use of oximetry ( Figure 5) it was found that sensitivity increased: 0.92 (95%CI, 0.87-0.95) and specificity remained constant: 0.98 (95%CI, 0.89-1.00). For the physical examination, a positive diagnostic likelihood ratio (DLR) of 46.2 (95%CI, 15.2-140.2), and a negative DLR of 0.47 (95%CI, 0.26-0.85) were obtained. Regarding physical examination plus oximetry, a positive diagnostic likelihood ratio (positive DLR) of 43.7 (95%CI, 8.0-239.8), and a negative diagnostic likelihood ratio (negative DLR) of 0.08 (95%CI, 0.05-0.14) were found.

### Statistical assessment of extracted data

**Figure 6.**
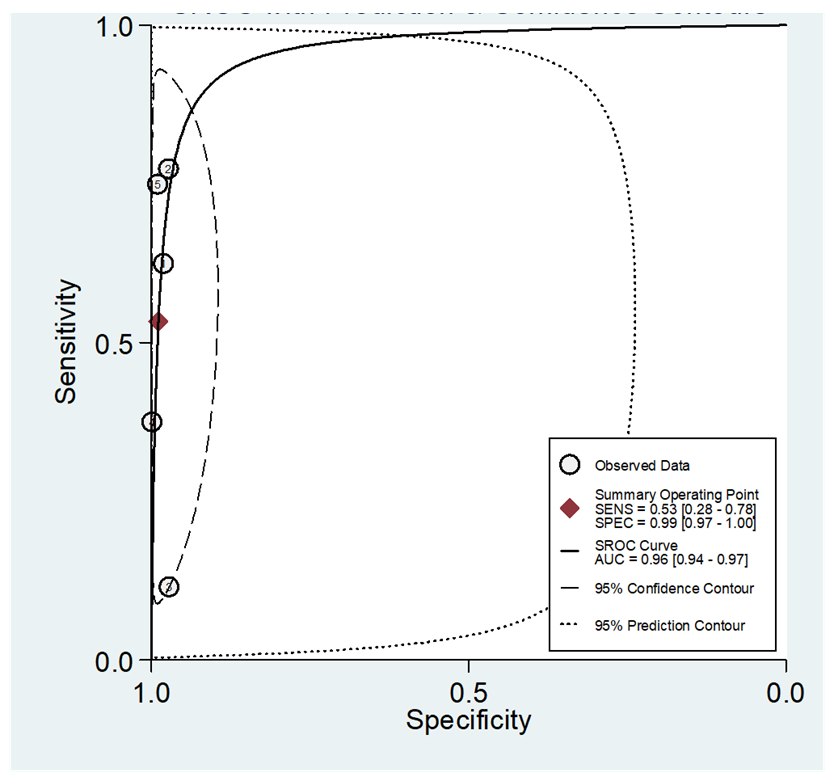
Receiving operating characteristic curve (ROC) showing the sensitivity and specificity graph for the physical examination as a screening test to detect critical congenital heart disease in asymptomatic newborns.

**Figure 7.**
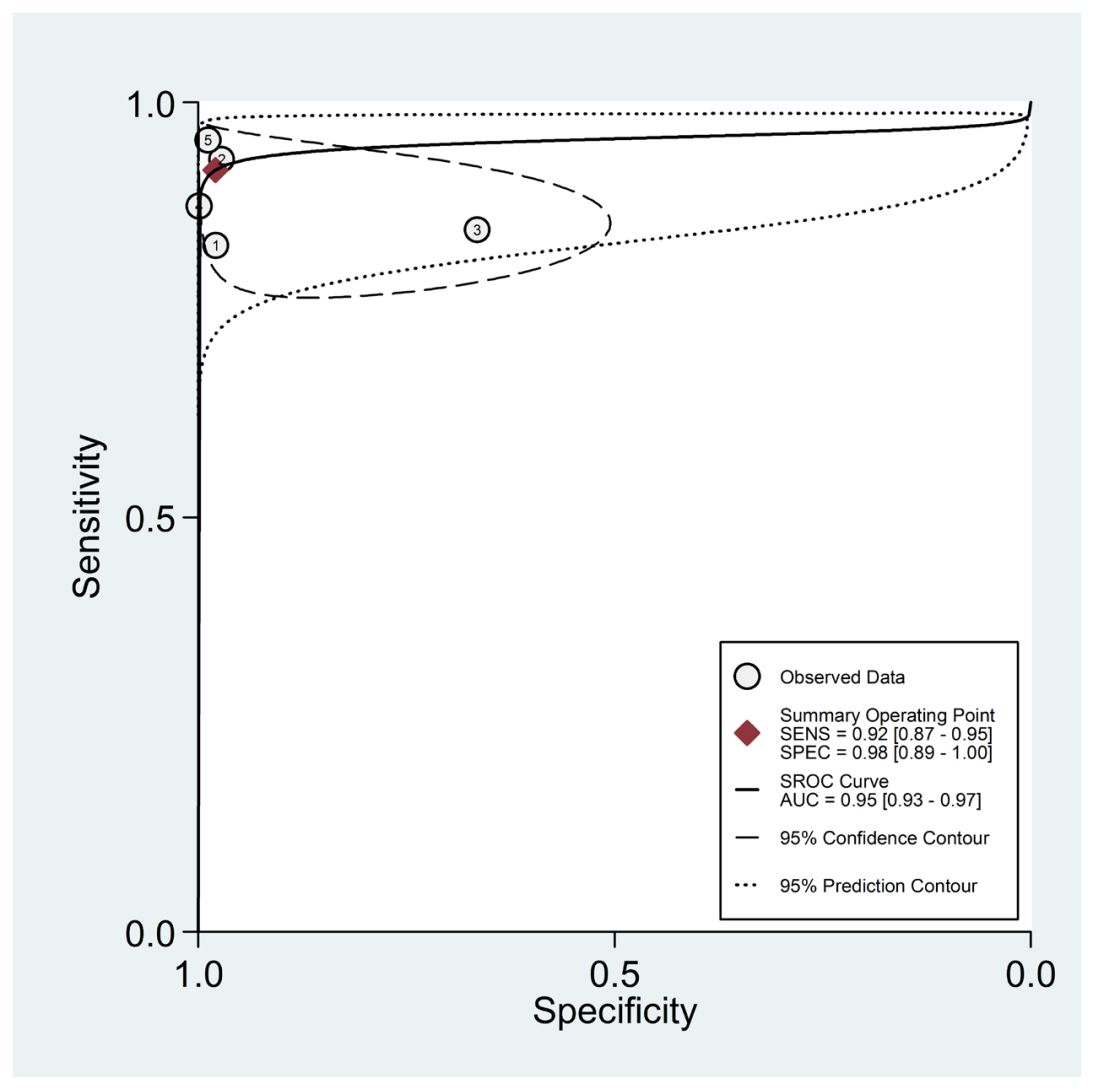
Receiving operating characteristic curve (ROC) showing sensitivity and specificity graph for the physical examination in combination with oximetry as a screening test to detect critical congenital heart disease in asymptomatic newborns.

Figure 6 and Figure 7 show the receptor operative characteristics (ROC) area under the curve (AUC) for physical examination, and physical examination plus oximetry. The AUC value found was 0.96 (95%CI, 0.94-0.97) for the physical exam, and 0.95 (95%CI, 0.93-0.97) when combining physical examination and oximetry, showing a similar diagnostic accuracy for both screening strategies. The following limits have been suggested to establish a diagnostic accuracy as per the AUC values as follows: low accuracy: AUC >0.5 and <0.7, moderate accuracy: AUC >0.7 and <0.9, and high accuracy: AUC >0.9 and ≤1 ^25^.

An I ^2^ of 94.91 (95%CI, 91.9-97.91) was found for the sensitivity of the physical examination, and an I ^2^ value of 82.42 (95%CI, 69.23-95.61) was found for the sensitivity of the physical examination in combination with oximetry. Likewise, the heterogeneity proportion, probably due to the threshold effect, was high (1.00), indicating the presence of a diagnostic threshold effect on the performance of the physical examination and oximetry screening.

### Risk of bias

**Figure 8.**
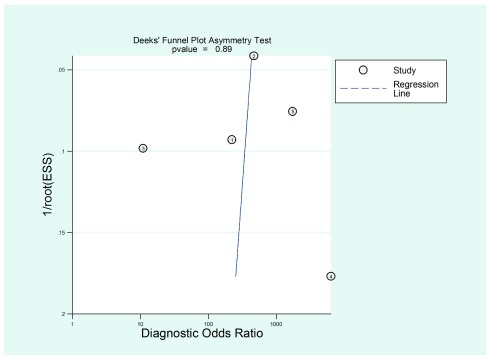
Deeks’ linear regression asymmetry test to assess publication bias.

Publication risk of bias was assessed through the Deeks’ regression test ^21^. Upon performing this evaluation, a non-statistically significant value was found for the coefficient corresponding to the slope (p = 0.89), which suggests symmetry in the data, and hence, a low probability of publication bias ( Figure 8).

### Clinical impact

**Figure 9.**
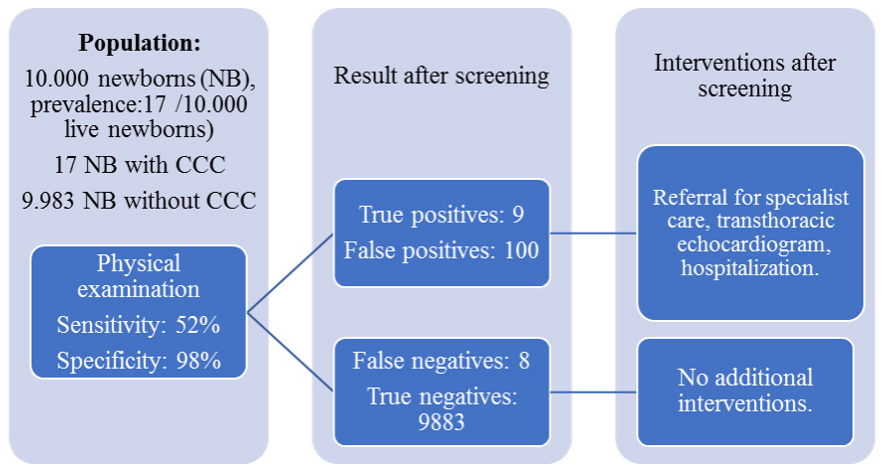
CCHD screening with physical examination on a presumptive cohort of 10,000 live NBs.

**Figure 10.**
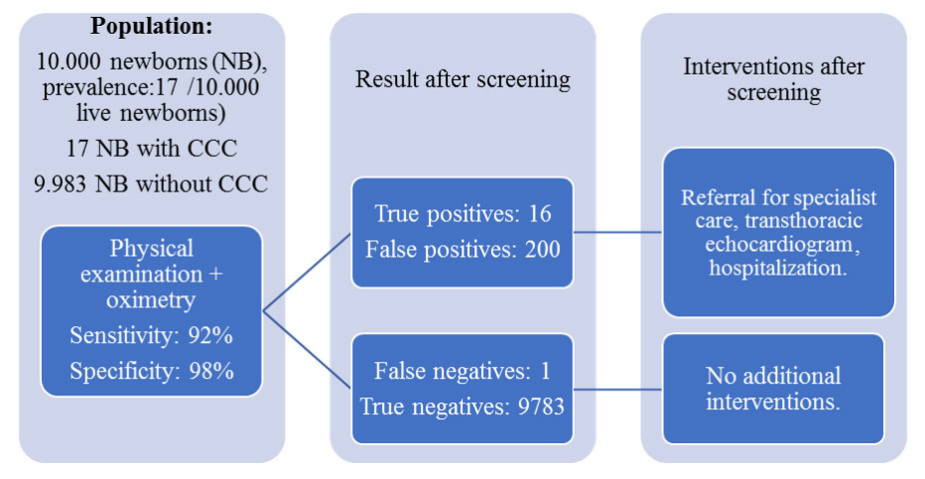
CCHD with physical examination + oximetry screening on a presumptive cohort of 10,000 live NBs.

Figure 9 shows an example of the results that would be obtained after the application of the physical examination on a hypothetical cohort of 10,000 live asymptomatic NBs and an expected prevalence of 17 cases of CCHD per 10,000 NBs ^2, 26^. Figure 10 shows the results that would be obtained in the same cohort when adding the use of oximetry to physical examination, it can be seen how the number of cases diagnosed in hospital increases almost twofold, and in a proportionate number, the number of false positive results increase.

## Discussion

CCHD represents a considerable cause of morbidity and mortality in newborns. Their early diagnosis has become an essential objective at the time of implementing a screening strategy involving oximetry, an aspect of the utmost importance, as late detection is associated with neurological and cognitive sequels in the NB, in addition to cardiac sequels, and social and economic repercussions.

The medical literature recommends the detection of CHD classified as "life-threatening" due to the risk of collapse and long-term sequels in the development of the NB ^27^. Among the CCHD that can be diagnosed through oximetry screening we find the following: interrupted aortic arch, coarctation of the aorta, dextro-transposition of the great arteries, double outlet right ventricle, Ebstein’s anomaly, hypoplastic left heart syndrome, pulmonary atresia, single ventricle, tetralogy of Fallot, total anomalous venous return, tricuspid atresia and truncus arteriosus.

Thanks to the inclusion of recent studies, this review tends to validate the importance of oximetry as a screening tool for this type of cardiopathies through the definition of their main operative characteristics. The data described in this study suggest that the physical examination in isolation does not offer reasonable levels of sensitivity for the diagnosis of CCHD in NBs, a feature observed in most of the studies. However, the specificity of the physical examination was high, a fact that was clinically expected. According to our data, when complementing the physical examination with oximetry, the sensitivity of the screening process is notably higher. The likelihood values described also tend to favor the complementary use of oximetry.

The degree of heterogeneity observed among the different study estimates is statistically significant; this variability might be explained as a result of the different cut-off points of "threshold effect", a finding that has been described as one of the most frequent primary causes of heterogeneity in meta-analyses of diagnostic tests. It occurs when differences in sensitivity and specificity are the result of there being different cut-off points or thresholds ^22^. As previously explained, the proportion of heterogeneity in this study is probably due to the threshold effect.

Another cause for the heterogeneity found in our study may be attributed to the different sample sizes found, which may also condition the presence of variability between studies; although this variation is meaningful from the statistic perspective, its clinical importance regarding diagnostic performance of physical examination and oximetry is objectionable, due to the operative characteristics already described and its diagnostic accuracy in the detection of CCHDs.

Likewise, the low sensitivity of physical examination found in most of the included articles may also influence the global estimate for heterogeneity. Among the causes for these low sensitivity values, the one provided by Saxena *et al*. ^23^ stands out, as it reports lower sensitivity values as the severity of the heart disease increases; they also report technical and human types of errors when conducting the screening, which might also have an effect on these low sensitivity figures.

Regarding the clinical usefulness of including oximetry in CCHD screening, we may conclude that it contributes to the early diagnosis of few cases, thus reducing the number of false negative results of the physical examination. According to Figure 9, it may be observed that 100 out of 109 NB with a positive screening would correspond to false positives and 9 would be true positives; out of the 9,891 NBs with a negative screening, eight would be false negatives with a form of CCHD, but they would not be detected by routine physical examination alone.

Furthermore, when adding the use of oximetry ( Figure 10), the number of cases diagnosed in hospital increases almost twofold, and in a proportionate number, the number of false positive results increase. Similarly, oximetry screening in addition to the physical examination reduces the number of NB with false-negative results ( Figure 10), going from 8 NBs with CCHDs that would not have been detected by the physical examination alone, to 1 NB when using physical examination in combination with oximetry. Although the number of false positives increases when oximetry is added, we consider this aspect as minor as compared with the reduction in the number of false negatives resulting from the application of screening, which would be reflected on better survival and in a reduction of the associated costs derived from additional medical and surgical interventions arising from a late diagnosis ^9^.

The above data are consistent with what previous studies have reported ^31^, whereby oximetry as an additional tool to the physical examination provides a timely diagnosis for almost 30 additional cases of CHD per 100,000 live NBs as compared to the use of the physical examination alone. At the same time, oximetry is considered a potentially efficient and appropriate tool to identify cases of CCHD that would otherwise go undetected after the physical examination of the NB ^31, 32^.

This review also confirms the finding described in a prospective multicenter study ^24^, but it is worth highlighting that the same study reports a global rate of false positives of less than 1% in the detection of CCHD; however, in this study the rate of false positives was affected by the time at which the oximetry was conducted, as it was significantly lower when the screening was conducted 24 hours after birth, as compared to those conducted before the 24 hours.

Among the strengths of this review, we may highlight the rigorous search conducted on the recent literature, and the standardized quality assessment performed on the articles included. Among the limitations we may include the small number of studies assessing the use of oximetry together with physical examination as a screening strategy for the detection of CCHD, and also the fact that one single evaluator conducted the assessment for selection, quality measurement, and data extraction, although formats previously standardized for this process were used.

## Conclusions

From this SLR and meta-analysis, it may be concluded that the use of oximetry, added to the conventional physical examination helps to detect a more significant number of NBs with CCHDs, without significantly increasing the number of false-positive results, a finding that may reduce the morbidity and mortality associated with hospital discharge of NB without a timely diagnosis ^33^.

This review also provides updated information which sets the bases for determining whether the impact of including this noninvasive technology as part of NB screening is cost-effective in low- and middle-income countries.

## Data availability

All data underlying the results are available as part of the article and no additional source data are required.

## Reporting guidelines

A completed PRISMA checklist is available from Open Science Framework: PRISMA checklist. DOI: https://doi.org/10.17605/OSF.IO/9SNCQ ^14^.
