# Supplementary material for: Oximetry and neonatal examination for the detection of critical congenital heart disease: a systematic review and meta-analysis
Source: F1000Res. 2019 Mar 1;8:242. [Version 1] doi: 10.12688/f1000research.17989.1 (PMC6659768; doi:10.12688/f1000research.17989.1)
Supplement: Peer review history

# Peer review: Reviewer response for version 1

**DOI:** 10.5256/f1000research.19674.r51065

**Published:** 2019-07-24

**Authors:** Praveen Kumar

**Affiliations:** 1 Department of Pediatrics, University of Illinois College of Medicine at Peoria, Peoria, IL, USA

Bello *et al.* completed a systematic review and meta-analysis to evaluate if pulse oximetry screening improves the diagnostic accuracy over physical examination alone for early detection of CCHD. Authors conclude that pulse oximetry in addition to neonatal physical examination improves sensitivity and may be particularly helpful in early detection of CCHD in low-resource settings.

 Authors have used appropriate search and statistical tools to complete their review. The manuscript is well written and easy to follow.

 Please correct the typing error at the end of introduction section - authors state their objective as "to define ..... CCHD in NB **younger than 37 weeks** without suspicion or prior diagnosis of CCHDs" but the review is based on data collected on term infants.

 Authors provide no data to support their conclusion that pulse oximetry is likely to be more helpful in early detection of CCHD in low-resource settings.

I confirm that I have read this submission and believe that I have an appropriate level of expertise to confirm that it is of an acceptable scientific standard.

# Peer review: Reviewer response for version 1

**DOI:** 10.5256/f1000research.19674.r48727

**Published:** 2019-06-07

**Authors:** Andrew K Ewer

**Affiliations:** 1 Institute of Metabolism and Systems Research, College of Medical and Dental Science, Neonatal Unit, Birmingham Women’s Hospital, Birmingham, UK

- A statistician will need to review the methodology and results. 
- I would remove the term 'in the prenatal stage' from the abstract, introduction and throughout.
- Include the recent Cochrane review on pulse oximetry screening. ^1^
- I do not understand the phrase ‘It is worth highlighting that there are no recent studies of this type which include the evidence available on oximetry screening in the newborn for the detection of CCHDs. Together with the use of oximetry,’. Please explain.
- P3: ‘in NB younger than 37 weeks’. Do you mean greater than 37 weeks?
- The PISMA Flow chart does not make sense as currently presented. Need to look again at the numbers coming of at each stage.
- ‘The echocardiogram was applied as a pattern of reference’ – This should be changed to gold standard test. 
- In 'Clinical impact', include numbers identified in both text and figure.

I confirm that I have read this submission and believe that I have an appropriate level of expertise to confirm that it is of an acceptable scientific standard.

## References

[1] : Pulse oximetry screening for critical congenital heart defects. *Cochrane Database of Systematic Reviews*.2018; 10.1002/14651858.CD011912.pub2 10.1002/14651858.CD011912.pub2 PMC649439629494750
